# Supplementary material for: Development of HER2-Specific Aptamer-Drug Conjugate for Breast Cancer Therapy
Source: Int J Mol Sci. 2020 Dec 21;21(24):9764. doi: 10.3390/ijms21249764 (PMC7767363; doi:10.3390/ijms21249764)
Supplement: Supplementary file 1 [file ijms-21-09764-s001.zip › Figure S1. Histochemistry of mouse tissues was performed using hematoxylin.docx]

**Supplementary Figure 1**. Histochemistry of mouse tissues was performed using the hematoxylin and eosin staining method. At the end of in vivo study, the tissues of tumor, heart, liver, lung and spleen taken from the all groups of mice were fixed and then stained in a hematoxylin and eosin staining solution. Images were taken at 400× magnification.
