# Supplementary material for: Development of HER2-Specific Aptamer-Drug Conjugate for Breast Cancer Therapy
Source: Int J Mol Sci. 2020 Dec 21;21(24):9764. doi: 10.3390/ijms21249764 (PMC7767363; doi:10.3390/ijms21249764)
Supplement: Supplementary file 1 [file ijms-21-09764-s001.zip › Table S1. Nucleic acid sequence information.docx]

Supplementary Table 1. Nucleic acid sequence information

| Nucleic acids | 5’ – Sequence – 3’ | Note |
| --- | --- | --- |
| HER2 DNA template | TAA TAC GAC TCA CTA TAG GGA GCC GCG AGG GGA GGG ATA GGG TAG GGC GCG GCT | T7 promoter binding site-inserted template |
| HER2 RNA aptamer | AGC CGC GAG GGG AGG GAU AGG GUA GGG CGC GGC U | For amplification of T7 promoter binding site-inserted ssDNA |
| Forward primer (T7) | TAA TAC GAC TCA CTA TA | For qPCR analysis |
| Forward primer | AGC CGC GAG GGG AGG |  |
| Reverse primer | AGC CGC GCC CTA CCC |  |
